# Supplementary material for: The Role of Flagellum and Flagellum-Based Motility on Salmonella Enteritidis and Escherichia coli Biofilm Formation
Source: Microorganisms. 2024 Jan 23;12(2):232. doi: 10.3390/microorganisms12020232 (PMC10893291; doi:10.3390/microorganisms12020232)
Supplement: Supplementary file 1 [file microorganisms-12-00232-s001.zip › microorganisms-2813859-supplementary.pdf]

Table S1. Analysis of the surface properties (PP, Glass and Steel). Contact angle (in degrees), with water (θ_W), formamide (θ_F), α-bromonaphtalene (θ_B); surface tension parameters(mJ/ m^2) and free energy of interaction (ΔG_{SWS}^{TOT})of the support materials (s) when immersed in water (w). Values are means ± SDs [22].

**Support Material**	**Contact Angle (°)**			**Surface Tension Parameters (mJ/m²)**			**Hydrophobicity (mJ/m²)**
	θ_W	θ_F	θ_B	γ_b^{LW}	γ_b^{+}	γ_b^{-}	ΔG_{SWS}^{TOT}
**PP**	107 ± 3.0	91.8 ± 2.4	53.2 ± 2.3	28.4	0	1.7	−76.6
**Glass**	73.5 ± 3.1	68.9 ± 3.1	50.8 ± 1.6	29.6	0	20	−13.8
**Steel 304**	101 ± 2.0	84.4 ± 2.0	46.5 ± 1.3	31.7	0	2.6	−71.5
